# Titania Nanotubes Grown on Carbon Fibers for Photocatalytic Decomposition of Gas-Phase Aromatic Pollutants

**DOI:** 10.3390/ma7031801

**Published:** 2014-03-04

**Authors:** Wan-Kuen Jo, Joon Yeob Lee, Ho-Hwan Chun

**Affiliations:** 1 Department of Environmental Engineering, Kyungpook National University, Daegu 702-701, Korea; E-Mail: jlee2@knu.ac.kr; 2 Department of Naval Architecture and Ocean Engineering, Pusan National University,Busan 609-735, Korea; E-Mail: chunahh@pusan.ac.kr

**Keywords:** titania loading, one-dimensional nanostructured, sub-ppm aromatic, BTEX

## Abstract

This study aimed to prepare titania (TiO_2_) nanotube (TNT) arrays grown on un-activated carbon fibers (UCFs), with the application of different TiO_2_ loadings based on the coating-hydrothermal process, and to evaluate their photocatalytic activity for the degradation of sub-ppm levels of aromatic pollutants (benzene, toluene, ethyl benzene, and o-xylene (BTEX)) using a plug-flow photocatalytic reactor. The characteristics of the prepared photocatalysts were determined by scanning electron microscopy (SEM),energy-dispersive X-ray (EDX), transmission electron microscopy (TEM), UV-visible absorption spectroscopy (UV-Vis) and X-ray diffraction (XRD) analyses. Spectral analysis showed that the prepared photocatalysts were closely associated with the characteristics of one-dimensional nanostructured TiO_2_ nanotubes for TNTUCFs and spherical shapes for TiO_2_-coated UCF (TUCF). The photocatalytic activities of BTEX obtained from TNTUCFs were higher than those obtained from a reference photocatalyst, TUCF). Specifically, the average degradation efficiencies of BTEX observed for TNTUCF-10 were 81%, 97%, 99%, and 99%, respectively, while those observed for TUCF were 14%, 42%, 52%, and 79%, respectively. Moreover, the photocatalytic activities obtained for TNTUCFs suggested that the degradation efficiencies of BTEX varied with changes in TiO_2_ loadings, allowing for the optimization of TiO_2_ loading. Another important finding was that input concentrations and air flow rates could be important parameters for the treatment of BTEX, which should be considered for the optimization of TNTUCFs application. Taken together, TNTUCFs can be applied to effectively degrade sub-ppm levels of gas-phase aromatic pollutants through the optimization of operational conditions.

## 1. Introduction

Specific structural designs of titania (TiO_2_) photocatalysts can be applied to enhance TiO_2_ oxidation activity for the treatment of a number of environmental pollutants [1]. Especially, one-dimensional structured TiO_2_ nanotube (TNT) arrays have unique charge-transport natures, large adsorption capacities, and multiple pathways for the enhanced diffusion of molecules [1–3]. The free movement of charge carriers over the length of TNTs can reduce the recombination rates of photo-induced electron and hole pairs when compared to TiO_2_ particles [3,4]. TNTs also contain large amounts of hydroxyl functional groups on the interlayer region of their walls, which are likely responsible for the reduced charge recombination rates [5]. In addition, TNTs are known to possess large surface areas and multiple channels for adsorption, resulting in a high adsorption capacity and improved photocatalytic performance [2]. These characteristics of one-dimensional TNTs have led to their applications to a number of environmental pollutant treatments. Several studies [4–6] have found that the photocatalytic performance of TNTs was superior to that of anatase phase TiO_2_ particles for the photocatalytic decomposition of a range of environmental pollutants such as aqueous phenols, methyl orange, and gaseous acetone.

Application of nanomaterials, including one-dimensional nanostructured TNTs and TiO_2_ nanoparticles, for air pollutant treatments requires their combination with a supporting substrate in order to minimize their escapes from photocatalytic reactors with cleaned air. Popular supporting substrates of nano-sized photocatalysts which have been applied to air purification include granular activated carbon [7], activated carbon fiber [8], glass materials [9], polymer materials [10], andun-activated carbon fiber [11,12]. Special attention has been given to un-activated carbon fibers (UCFs) as photocatalyst supporting materials because of their unique properties, such as temperature and corrosion resistance, and high strength and flexibility [12]. The combination of TiO_2_ with carbon materials can also retard the recombination rates of photo-generated electron and positive hole pairs, thereby enhancing photocatalytic activity [13]. In addition, UCFs are quite robust, and hence, are resistant to degradation throughout photocatalysis, whereas some other polymers or cotton textiles are less durable [11,14]. Nevertheless, to date, only limited studies of the application of TiO_2_ combined with UCFs have been carried out [11,12]. Moreover, the aforementioned studies investigated the photocatalytic activity of TNT or TiO_2_ nanosheet arrays grown on UCFs based on the degradation of aqueous-phase dyes such as methyl orange and rhodamine B. The photon absorbance mechanismsand heterogeneous photocatalytic kinetics of chemical species differ between the two different interfaces [15]. As such, there are still a number of relevant yet unanswered questions relating tothe photocatalytic performance of TNTs grown on UCFs, and their applications to treatment of polluted air.

In this study, TNT arrays on UCFs (TNTUCFs) with different loadings of the TiO_2_ source were prepared using a coating-hydrothermal process, and their photocatalytic activities were investigated for the decomposition of aromatic pollutants at indoor air concentration levels (sub-ppm). A TiO_2_-coated UCF (TUCF) was also investigated as a reference photocatalyst for its surface characteristics and photocatalytic decomposition under the same photocatalytic conditions. The target chemicals were four aromatic volatile organic compounds (VOCs) (benzene, toluene, ethyl benzene, and o-xylene, (BTEX)), which were selected based on their prevalence in indoor air [16] and due to their representing both carcinogenic and non-carcinogenic health hazards [17,18].

## 2. Results and Discussion

### 2.1. Characteristics of Prepared Photocatalysts

**Figure 1.**
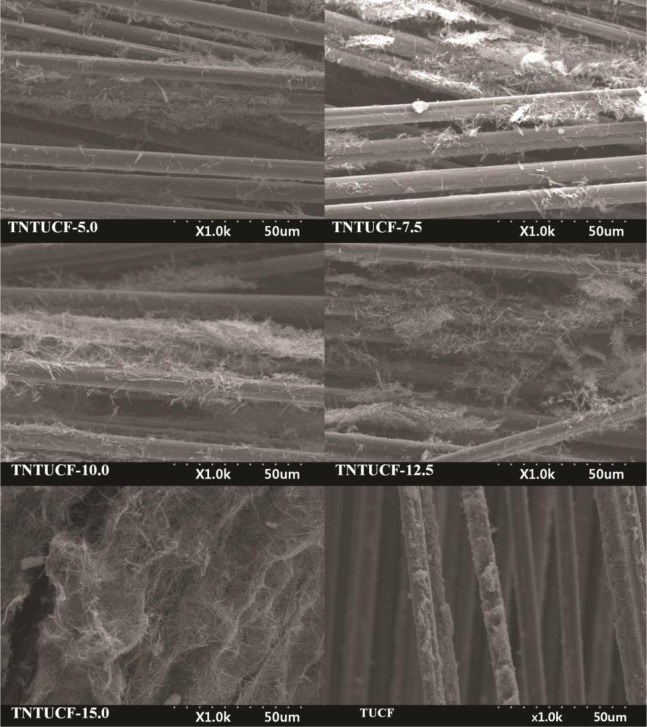
Scanning electron microscopy (SEM) of titania nanotube arrays grown on un-activated carbon fibers (TNTUCFs) with different TiO_2_ loadings (TNTUCF-5, TNTUCF-7.5, TNTUCF-10, TNTUCF-12.5, TNTUCF-15) and TiO_2_-coated UCF (TUCF).

**Figure 2.**
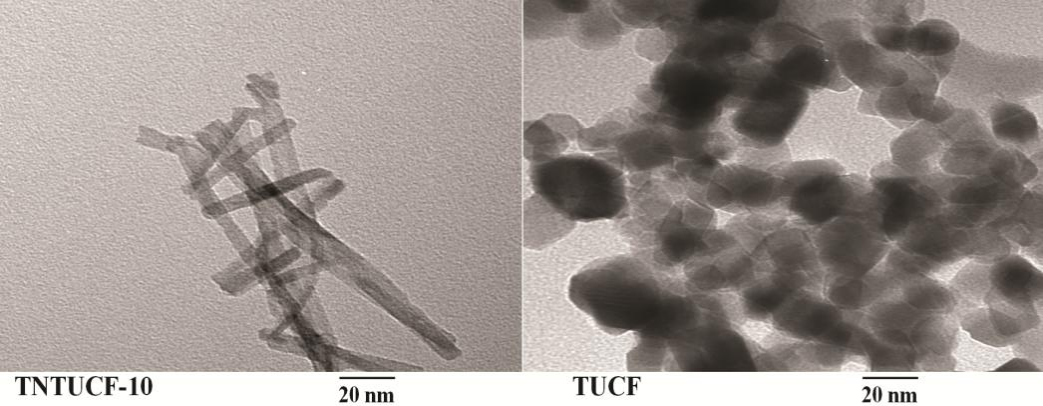
Transmission electron microscopy (TEM) of TNTUCF-10, and TUCF.

TNTUCFs with different TiO_2_ loadings, as well as a reference TUCF photocatalyst, were characterized by SEM, EDX, TEM, and XRD analyses. Figure 1 displays the SEM photographs of TNTUCF-5, TNTUCF-7.5, TNTUCF-10, TNTUCF-12.5, TNTUCF-15 and TUCF. The SEM images of TNTUCFs showed the TNT arrays grown on UCFs, of which the degree of cover was increased with increasing TiO_2_ loading, whereas SEM images of TUCF exhibited TiO_2_ nanoparticles coated onto UCFs. In addition, the TEM images of TNTUCF-10 revealed the nanotubular structure, whereas those for TUCF showed spherical shapes (Figure 2). Similarly, Fu *et al*. [19] reported that an N-TiO_2_ structure prepared using a solid-state reaction method with NH_4_Cl as the nitrogen source revealed a tubular structure in its TEM image. For the present study, the length and outer diameter of the TNTUCF-10 were *ca.* 20–110 nm and *ca*. 6–11 nm, respectively. Consequently, these results demonstrated that the hydrothermal process used in this study could be used to grow TNTs on the surface of the supporting substrate (UCF) to form TNTUCFs. In addition, Guo *et al*. [12] reported that TiO_2_ nanosheet arrays could be successfully grown on UCFs via an alternative hydrothermal process.

**Figure 3.**
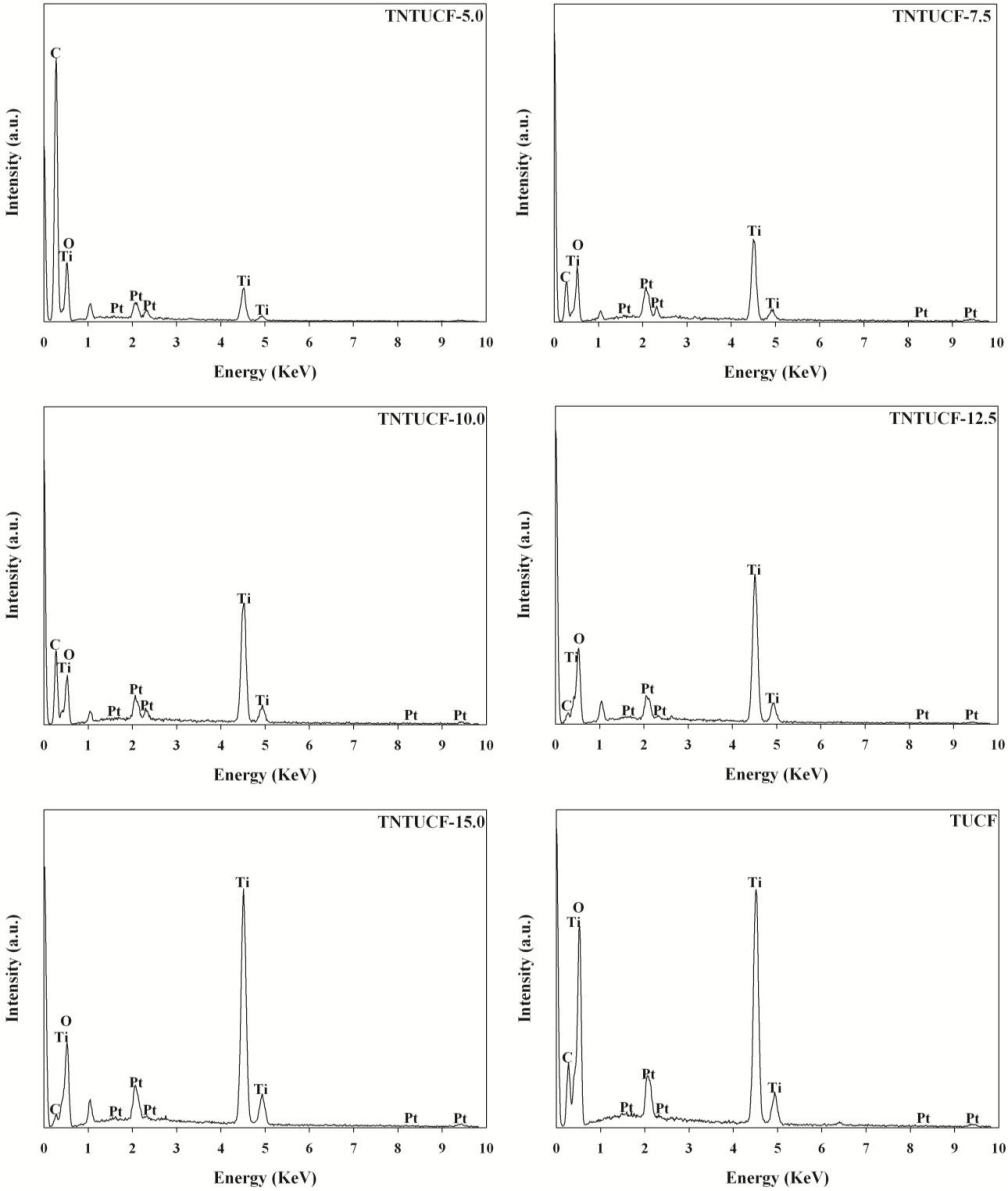
Energy-dispersive X-ray (EDX) of five TNTUCFs with different TiO_2_ loadings (TNTUCF-5, TNTUCF-7.5, TNTUCF-10, TNTUCF-12.5, TNTUCF-15) and TUCF.

The EDX spectra of the TNTUCFs displayed peaks of C, Ti, Pt, and O atoms with different peak intensities (Figure 3). The peaks of the Ti and O atoms were assigned to TiO_2_, while the C atom peak was attributed to UCF. In addition, Pt atoms were considered to have originated from the Pt coating pretreatment of the TNTUCF samples prior to conducting SEM analysis. Similar to the TNTUCFs,the EDX spectra of the TUCF also revealed peaks of C, Ti, Pt, and O atoms, although their peak intensities differed.

**Figure 4.**
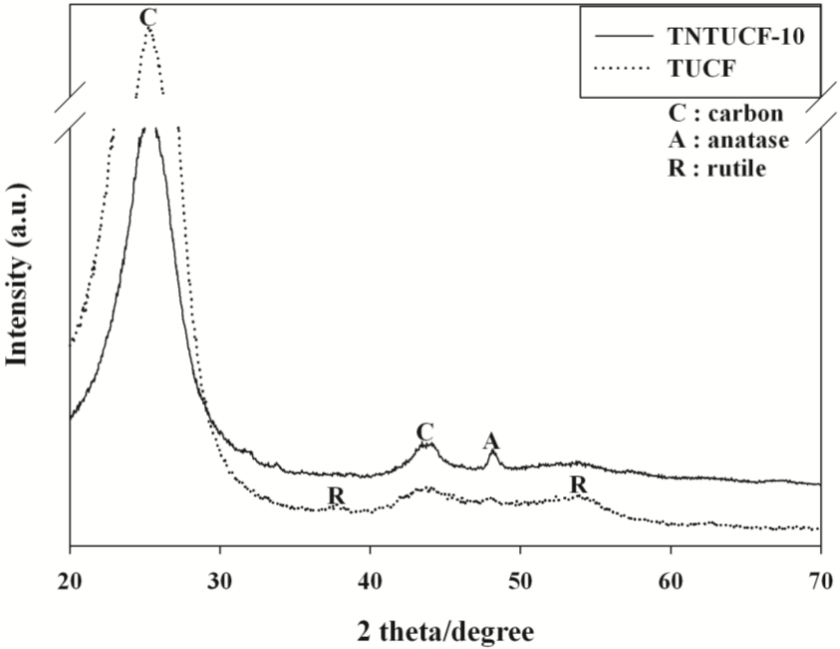
X-ray diffraction (XRD) patterns of a representative TNTUCF (TNTUCF-10) and TUCF.

Figure 4 illustrates the XRD images of TNTUCF-10 and TUCF. Both photocatalysts showed a broad C peak at 2θ = 25.3° and a smaller C peak at 2θ = 43.3°. In addition, TNTUCF-10 revealed an anatase crystal phase with a peak at 2θ = 48.2°, while the typical anatase peak at 2θ = 25.3° was thought to be overlapped with the carbon peak of UCFs. These results were similar to those obtained by Chen *et al*. [11]. Accordingly, the prepared one-dimensional structured TNTs had the characteristics of TiO_2_ crystals, which have photocatalytic activity for the degradation of environmental pollutants. In contrast, TUCF showed rutile crystal phases with two peaks at 2θ = 37.6° and 53.9°, while the typical anatase peak at 2θ = 25.3° and rutile peak at 2θ = 27.4° were thought to be overlapped with the carbon peak of UCFs. These results were ascribed to the uses of Degussa P25 TiO_2_ for the preparation of TUCF, which is composed of both rutile and anatase phases. Although the UV-Vis spectra of the prepared photocatalysts were not shown in this paper because of limited space, their band gap energies estimated using their UV-Vis absorption spectra were all close to 3.1 eV.

### 2.2. Photocatalytic Activity of As-Prepared Photocatalysts

**Figure 5.**
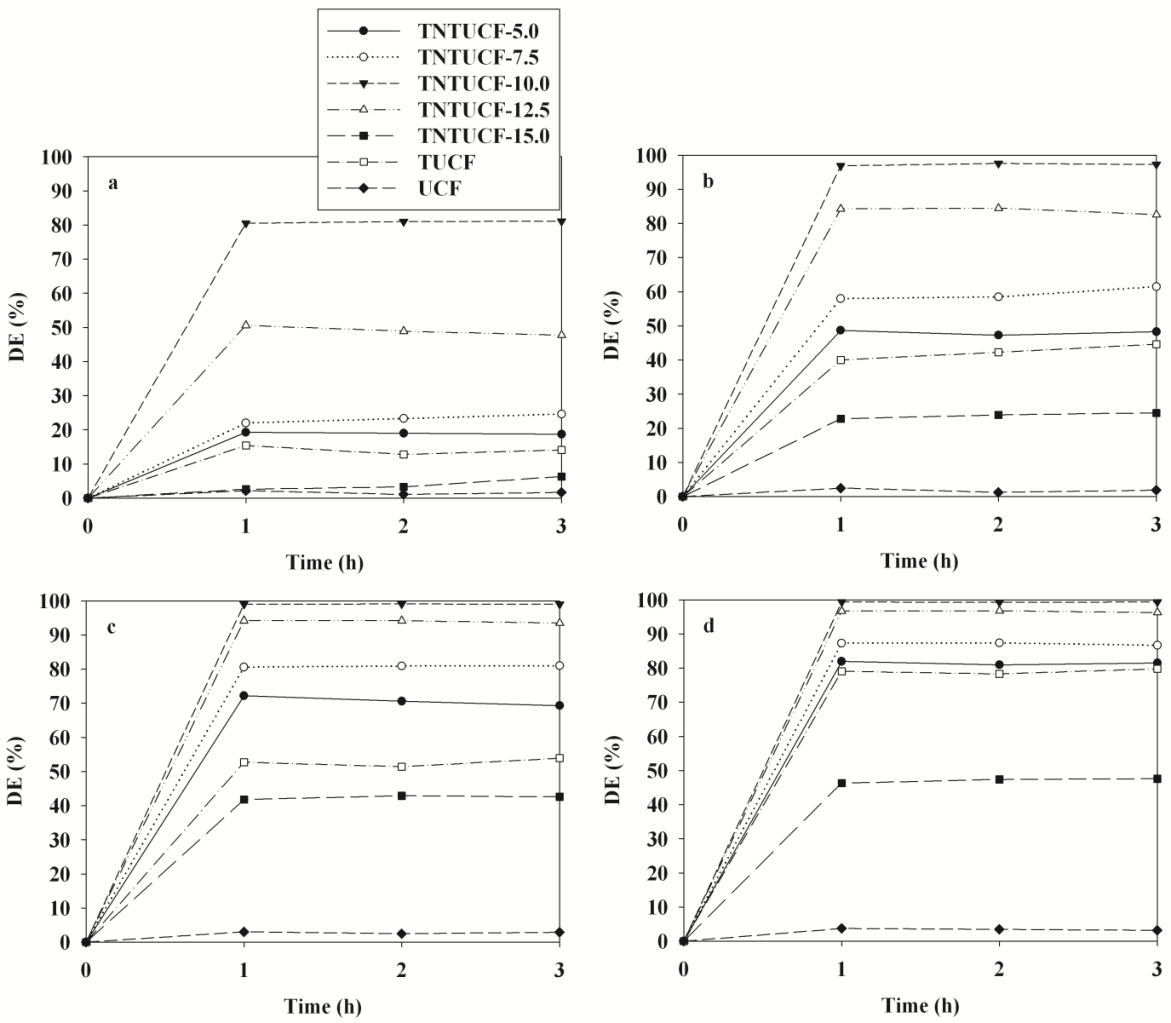
Degradation efficiencies (DE) of (**a**) benzene; (**b**) toluene; (**c**) ethyl benzene;(**d**) o-xylene, as determined using TNTUCFs with different TiO_2_ loadings (TNTUCF-5, TNTUCF-7.5, TNTUCF-10, TNTUCF-12.5, TNTUCF-15), TUCF, and UCF under UV light irradiation.

The photocatalytic activity of four TNTUCFs, TUCF, and UCF were evaluated based on their ability to decompose four target compounds (BTEX). Figure 5 shows the time-series degradation efficiencies of BTEX observed via the as-prepared photocatalysts. All BTEX degradation efficiencies determined for the TNTUCFs were higher than those of TUCF and UCF, except for those of TNTUCF-15. Specifically, the average degradation efficiencies of BTEX for the TNTUCF-10 were 81%, 97%, 99% and 99%, respectively, while those for TUCF were 14%, 42%, 52% and 79%, respectively. Additionally, the BTEX removal efficiencies determined via the TNTUCF-10 were higher than those obtained via a prototype photocatalyst (P25 TiO_2_), which were reported inJo *et al*. [10]. Specifically, the aforementioned study reported that the average degradation efficiencies of BTEX determined for P25 TiO_2_ under similar operational conditions were 28%, 38%, 51%, and 64%, respectively. The higher degradation efficiencies of TNTUCFs were attributed to synergistic effects between their large adsorption capacity and reduced recombination rates of photo-induced electron and hole pairs of one-dimensional nanostructured TNTs [1–3]. Moreover, the BTEX degradation efficiencies determined for the TNTUCFs increased as the TiO_2_ loadings increasedfrom 5% to 10%, but then decreased as the TiO_2_ loadings increased further from 10% to 15%. Specifically, the average degradation efficiencies of BTEX for the TNTUCF-5 were 19%, 48%, 71%, and 81%, respectively, while those for TNTUCF-15 were 4%, 24%, 42%, and 47%, respectively. These findings allowed for the optimization of TiO_2_ loading during TNTUCFs preparation for BTEX degradation. For high TiO_2_ loading conditions, TiO_2_ nanoparticles might block some of carbon sites, lowering adsorption capacity of prepared photocatalysts. In a similar study, Chen *et al*. [11] reported that TNTUCF showed a higher photocatalytic efficiency for the degradation of aqueous rhodamine B when compared with pure UCF. In addition, Guo *et al*. [12] showed that TiO_2_ nanosheets grown on carbon fibers had higher photocatalytic activities for aqueous methyl orange when compared with pure UCF. Meanwhile, a long-term study is suggested to examine the dependence of the activity of the prepared photocatalysts on application time.

**Figure 6.**
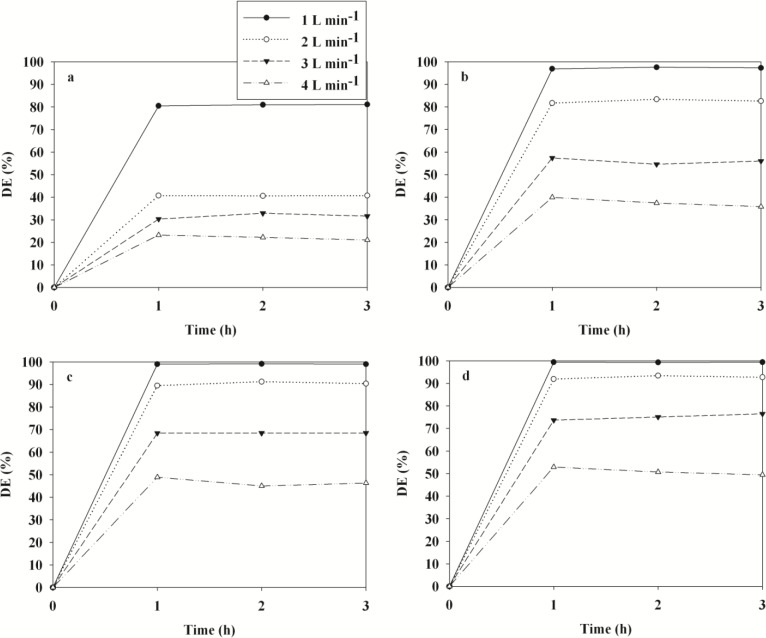
Degradation efficiencies (DE) of (**a**) benzene; (**b**) toluene; (**c**) ethyl benzene; (**d**) o-xylene as determined using TNTUCF-10 according to various air flow rates (1, 2, 3, and 4 L·min^−1^).

The time-series degradation efficiencies of BTEX obtained via the representative TNTUCF-10 according to air flow rates (AFRs) are shown in Figure 6. The BTEX degradation efficiencies revealed a decreasing trend with increasing AFRs. Specifically, the average degradation efficiencies of BTEX decreased from 81% to 22%, 97% to 37%, 99% to 46%, and 99% to 51%, respectively, as the AFR increased from 1 to 4 L·min^−1^. Similarly, Yu and Brouwers [20] reported a descending trend in NO degradation efficiencies within an AFR range of 1–5 L·min^−1^. These results were attributed to the mass transfer of target compounds to the surface of the catalysts and the reaction kinetics of target compounds, which are two important factors of heterogeneous photocatalytic degradation [21,22]. The mass transfer rate of gas-phase pollutants in a plug-flow reactor is strongly associated with the face velocities of air [23]. Accordingly, the degradation efficiencies of BTEX might have increased as face velocities increased, if the increased mass transfer rate was likely a crucial factor for the photocatalytic degradation kinetics of BTEX. Conversely, for the present study, the degradation efficiencies of BTEX decreased as the face velocities increased. Specifically, the face velocities for AFRs of 1, 2, 3, and 4 L·min^−1^ corresponded to 2.1, 4.2, 6.3, and 8.4 cm·s^−1^, respectively, suggesting that the degradation efficiencies of BTEX obtained from TNTUCF-10 would have been somewhat limited by reaction kinetics on the photocatalyst surface. This suggestion was supported by the finding that the residence times of the target compounds in the plug-flow reactor, which were calculated by dividing the reactor volume by the AFRs, decreased as the AFR increased. Specifically, the residence times for the AFRs of 1, 2, 3 and 4 L·min^−1^ were 16.4, 12.3, 8.2 and 4.1 s, respectively. Consequently, the low degradation efficiencies of BTEX under high AFR conditions were likely due to low reaction rates on the catalyst surface because of insufficient reaction times in the plug-flow reactor.

**Figure 7.**
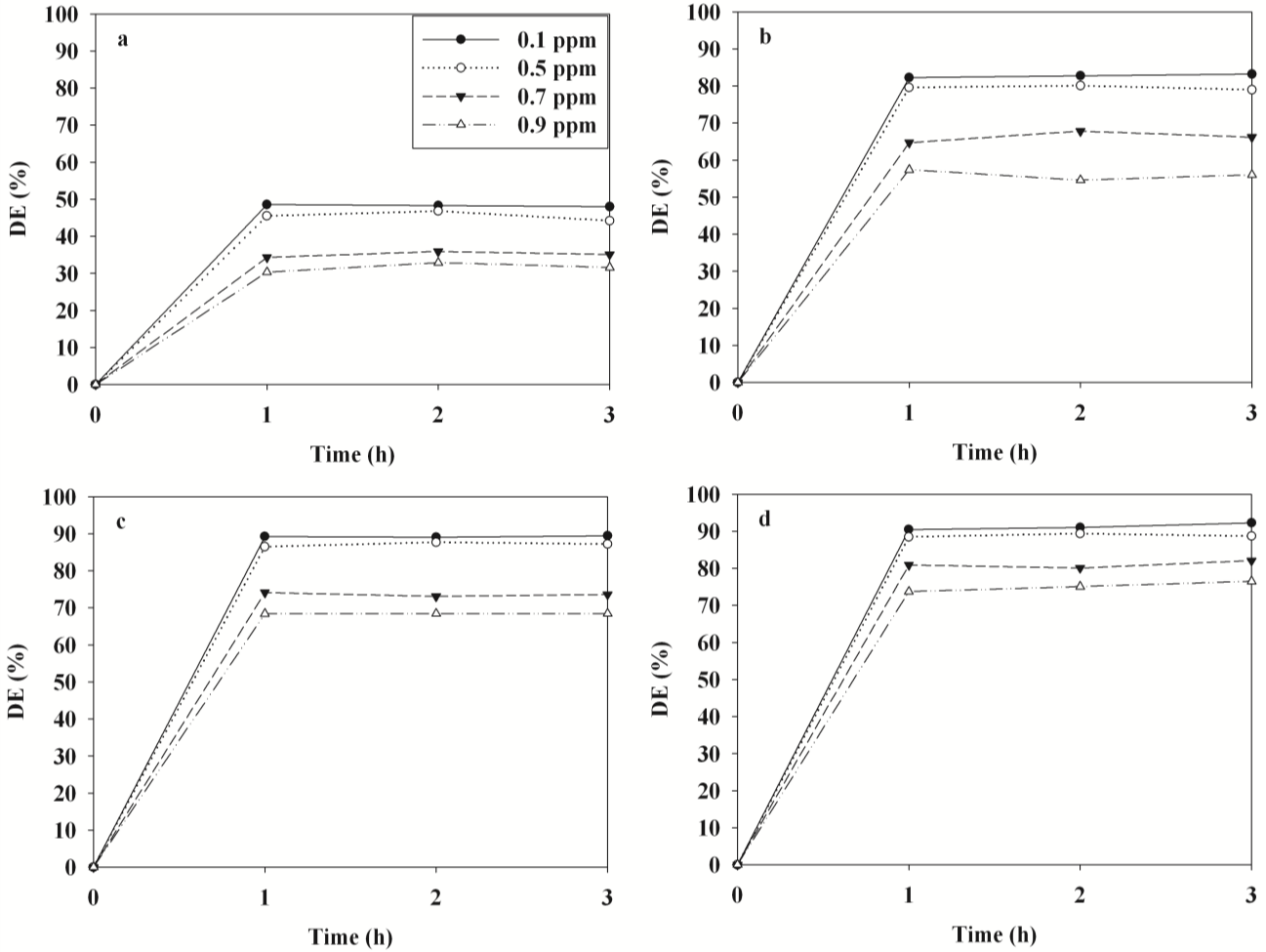
Degradation efficiencies (DE) of (**a**) benzene; (**b**) toluene; (**c**) ethyl benzene; (**d**) o-xylene determined using TNTUCF-10 according to varying input concentrations (0.1, 0.5, 0.7, and 0.9 ppm).

Figure 7 illustrates the time-series degradation efficiencies of BTEX determined via TNTUCF-10 according to input concentrations (ICs) within a range of typical indoor air concentrations. For the target compounds, the degradation efficiencies decreased as ICs increased. Specifically, at the lowest IC of 0.1 ppm the average degradation efficiencies of BTEX were 48%, 83%, 89% and 91%, respectively, whereas those for the highest IC of 0.9 ppm were 32%, 56%, 68% and 75%, respectively. Similarly, Devahasdin *et al*. [24] reported that the NO degradation efficiency determined using UV-activated TiO_2_ decreased from 70% to 15% when IC increased from 5 to 60 ppm. Yu and Brouwers [20] also found that the degradation efficiency of NO determined using visible light-activated carbon-impregnated TiO_2_ decreased from 61% to 16% when IC increased from 0.1 to 0.9 ppm. Because chemical adsorption onto photocatalyst surfaces is known as a primary factor influencing the degradation efficiency of chemicals [22], the increasing pattern in degradation efficiencies with IC increases was ascribed to adsorption competition of BTEX molecules on the catalyst surface. Specifically, a small amount of the active adsorption sites on the surface of the TNTUCF-10 under low IC conditions would have been available for BTEX adsorption before the surface reaction was initiated.

## 3. Experimental Section

### 3.1. Synthesis and Characterization of TUCF and TNTUCFs

A TUCF was synthesized using a simple coating process, while TNTUCFs with different TiO_2_ loadings were prepared by hydrothermally treating the prepared TUCF. CF sheets (Hyundai Fiber Co., Hanam, Korea) were first washed with NaOH solution (1 M, Sigma-Adrich, St. Louis, MO, USA) and then with distilled water, after which they were dried in an oven at 110 °C. The cleaned CFs were immersed into tetrabutyl titanate (TBT, Sigma-Aldrich, St. Louis, MO, USA)-hexane (Sigma-Aldrich, St. Louis, MO, USA) solution (10 vol% TBT) for 10 min and were then exposed to water-saturated air for the hydrolysis of TBT. These immersion and hydrolysis processes were repeated five times in order to obtain high TiO_2_ loaded CFs. The coated CF was calcined in an oven at 400 °C for 6 h to obtain TUCF. In addition, CF sheets were immersed in TBT-hexane solutions (5%, 7.5%, 10%, 12.5%, or 15%) to prepare TNTUCFs with different TiO_2_ loadings (named as TNTUCF-5, TNTUCF-7.5, TNTUCF-10, TNTUCF-12.5, or TNTUCF-15, respectively). To hydrothermally treat the coated CFs, they were rolled up and immersed into NaOH solution (10 M) in a stainless steel autoclave (170 mL) with Teflon-lined inner-walls, after which they were heated at 160 °C for 48 h. The autoclave was then cooled down to room temperature, and the hydrothermally treated samples were washed with acetic acid (0.1 M, Sigma-Aldrich, St. Louis, MO, USA) and distilled water in turn, after which they were calcined in an oven at 400 °C for 6 h to obtain final products, as white powders. The characteristics of the prepared TNTUCFs and TUCF were examined using scanning electron microscopy (FE-SEM, Hitachi S-4300, Tokyo, Japan), energy-dispersive X-ray (EDX, Hitachi EDX-350, Tokyo, Japan), transmission electron microscopy (TEM, Hitachi H-7600, Tokyo, Japan), and X-ray diffraction (XRD, Rigaku D/max-2500 diffractometer, Rigaku, Tokyo, Japan) spectroscopy. In addition, for the calculation of band gaps of the prepared photocatalysts, UV-Vis absorption spectra were obtained for the dry pressed disk samples using a Varian CARY 5G spectrophotometer (Vrian Inc., Cary, NC, USA) in the wavelength range between 200 and 800 nm at a scanning rate of 120 nm/min.

### 3.2. Photocatalysis of BTEX via TNTUCFs and TUCF

The photocatalytic activity of the TNTUCFs, TUCF, and UCF for the degradation of aromatic VOCs were examined using a continuous-flow Pyrex tubing reactor (4.0 cm inside diameter (i.d.) and26.5 cm length), the inner wall of which was covered with each of the prepared photocatalysts. A cylindrical UV-light source (8-W fluorescent black light lamp, F8T5BL, Youngwha Lamp Co., Seoul, Korea) was inserted inside the Pyrex reactor, serving as the inside surface boundary layer of the reactor. The high-purity dried air supplied by the air cylinder was further cleaned by passing through an activated carbon filter. Humidified air was generated by passing the cleaned air through a humidification device immersed in a water bath. Standard gases were produced by mixing the humidified air with the target chemicals injected into a heated buffering Pyrex bulb via anauto-controlled syringe pump (Model Legato 100, KdScientific Inc., Holliston, MA, USA). The produced standard gases were allowed to flow into another buffering Pyrex bulb to decrease the fluctuation of input concentration fluctuation prior to being fed into the photocatalytic reactor.

The photocatalytic activity tests of the prepared photocatalysts were conducted under different operational conditions by varying air flow rates (AFRs) and input concentrations (ICs). The AFRs were adjusted to 1.0, 2.0, 3.0, or 4.0 L·min^−1^, while ICs were adjusted to 0.1, 0.5, 0.7, or 0.9 ppm.For each parameter test, the other parameters were fixed to their representative values: AFR,1.0 L·min^−1^; and IC, 0.9 ppm. The relative humidity (RH) was fixed 45%, which is within the range of comfort (40%–60%) recommended by the American Society of Heating, Refrigerating and Air Conditioning Engineers. The hydraulic diameter (HD, defined as the inner diameter of the Pyrex tubing reactor minus the outside diameter of the light source) of the Pyrex reactor was 1.2 mm. The light intensity supplied by the light source was 0.4 mW·cm^−2^ at a distance from the light source equal to half the HD of the photocatalytic reactor. Each test was repeated three times in order to obtain more reliable results.

Measurements of gaseous chemicals were conducted both upstream and downstream of the Pyrex tubing reactor. Gas samples were collected by drawing air through a stainless steel tube (1/4 in. i.d. and 10 cm length) containing Tenax-TA adsorbent. The gaseous species collected on the Tenax-TA trap were analyzed by combining a thermal desorbing device (ATD 500, Perkin Elmer Co., Shelton, CT, USA) to a gas chromatograph (GC, Perkin Elmer Clarus 680, Shelton, CT, USA)/mass spectrometer (MS, Perkin Elmer Clarus SQ8 T, Shelton, CT, USA) equipped with a capillary column (DB-1, Agilent Co., Santa Clara, CA, USA). The trap was thermally desorbed at 250 °C for 10 min, and the gaseous species were cryofocussed at −30 °C using an internal cryo trap, after which they were rapidly heated to 250 °C and subsequently flushed to transfer the target compounds to the GC/MS system. The initial oven temperature was set to 40 °C for 5 min, and was then ramped to 230 °C at5 °C min^−1^ for 7 min. The gaseous species were identified based on their reaction times and mass spectra (Wiley 275 software library), while their quantification was carried out using calibration equations based on five different concentrations. The quality control program for gas measurements included blank and spiked sample traps. On each day, a blank sample was analyzed in order to examine the trap for contamination. A spiked sample was analyzed daily to validate the quantitative response of the analytical system. The method detection limits for BTEX ranged from 0.005 to0.008 ppm, depending on the chemical species.

## 4. Conclusions

In this study, TNTUCFs with different TiO_2_ loadings were prepared using a coating-hydrothermal process and their photocatalytic activities were investigated for the decomposition of sub-ppm levels of aromatic pollutants using a plug-flow photocatalytic reactor. Spectral results of the prepared photocatalysts were closely associated with the characteristics of one-dimensional nanostructured TNTs for TNTUCFs and spherical shapes for TUCF. TNTUCFs exhibited superior photocatalytic degradation of BTEX compared to that of TUCF. Moreover, the photocatalytic activities obtained from TNTUCFs suggested that the degradation efficiencies of BTEX varied with changes in TiO_2_ loading, allowing for the optimization of TiO_2_ loading. Another important finding was that ICs and AFRs could be important parameters for the treatment of sub-ppm level vapors, which should be considered for the optimum operation of TNTUCFs.
